# Rat eradication comes within a whisker! A case study of a failed project from the South Pacific

**DOI:** 10.1098/rsos.160110

**Published:** 2016-04-20

**Authors:** W. Amos, H. J. Nichols, T. Churchyard, M. de L. Brooke

**Affiliations:** 1 Department of Zoology, University of Cambridge, Downing Street, Cambridge, UK; 2 School of Natural Science and Psychology, Liverpool John Moores University, Liverpool, UK; 3 RSPB Centre for Conservation Science, Royal Society for the Protection of Birds, Sandy, UK

**Keywords:** brodifacoum, genetic diversity, Henderson Island, heterozygosity, Pitcairn Islands, *Rattus exulans*

## Abstract

To enhance their conservation value, several hundred islands worldwide have been cleared of invasive alien rats, *Rattus* spp. One of the largest projects yet undertaken was on 43 km^2^ Henderson Island in the Pitcairn group, South Pacific, in August 2011. Following massive immediate mortality, a single *R. exulans* was observed in March 2012 and, subsequently, rat numbers have recovered. The survivors show no sign of resistance to the toxicant used, brodifacoum. Using pre- and post-operation rat tissue samples from Henderson, plus samples from around the Pacific, we exclude re-introduction as the source of continued rat presence. Microsatellite analysis of 18 loci enabled comparison of genetic diversity of Henderson rats before and after the bait drop. The fall in diversity measured by allele frequency change indicated that the bottleneck (*N*_e_) through which the breeding population passed was probably around 50 individuals, representing a census population of about 60–80 animals. This is the first failed project that has estimated how close it was to success.

## 1. Introduction

Remote oceanic islands harbour a wide range of species found nowhere else in the world; yet, many of these species are being driven to extinction by the introduction of alien species, most notably rats, *Rattus* spp. [1,2]. Recent conservation efforts have led to rodent eradication operations on 719 islands across the world (http://diise.islandconservation.org/, accessed 10 April 2015). For larger islands, the eradication method of choice usually involves helicopter distribution of poison bait. The great majority, 80% (578 of 719), of such operations are successful but, despite meticulous planning, a minority fail and some correlates of failure are emerging [3]. A more refined understanding of the reasons behind failures will, in part, depend on knowing how close a project was to success. More specifically, in the aftermath of a failure, it would be valuable for conservation planners to know whether a mere handful of rats survived the poison bait, in which case minor tweaking of the baiting protocol might ensure the success of a second attempt, or whether survivors numbered hundreds or more, suggesting the need for major revision of the eradication protocol [4]. The study we report here is, to our knowledge, the first attempt to use genetic data to estimate the number of survivors of a failed rat eradication operation (but see [5]).

The project in question was undertaken in August 2011 on the 43 km^2^ World Heritage site of Henderson Island (24°20′S, 128°19′W) in the Pitcairn group, South Pacific. The island is home, inter alia, to four species of endemic landbird and four breeding species of surface-nesting *Pterodroma* petrel. The latter's chicks are known to be victims of predation by Pacific rats, *Rattus exulans*, which arrived with Polynesian colonists some 800 years ago [6,7]. To rid the island of rats, 75 tonnes of bait, laced with the anti-coagulant poison brodifacoum, were dropped from helicopters in an operation costing about £1.5 million organized by the Royal Society for the Protection of Birds (RSPB) [8]. Massive immediate mortality was achieved and no rats were seen on the island for the first three months when personnel were on-island continuously. However, in March 2012, seven months after the bait drop, a single *R. exulans* was observed by a temporary visitor. Since then rat numbers (which may vary with season) have recovered fully to 50 000–100 000 individuals [9–11].

Independent reviews of the project in 2013 did not identify any operational flaws [12]. However, in the absence of genetic data, the reviews could exclude neither re-invasion of rats from elsewhere nor brodifacoum resistance among Henderson rats.

By sampling rats from other island groups in the region, we confirmed that the project's failure was indeed a failure of eradication as opposed to an untimely re-introduction, from neighbouring islands [13]. Furthermore, a comparison of microsatellite data from rats sampled before the operation, in 2009, and after, in 2012 and 2013, allowed us to estimate the number of animals to which the population was reduced. Finally, on-island tests of the survivors in 2013 yielded no evidence that the failure was due to brodifacoum resistance among those survivors.

## 2. Material and methods

### 2.1. Study sites and sample acquisition

Henderson Island is roughly 9 km north-to-south by 5 km east-to-west. All rat samples were obtained in the northern one-third of the island (electronic supplementary material, figure S1) because movement over the terrain is so difficult for people due to the thickness of the vegetation.

However, the vegetation would pose no barrier to a rat, and there are no obvious topographical features that would impede rat dispersal across the island. Most of the samples were obtained by snap-trapping, either on the plateau, a former lagoon on this raised coral island now lying 30 m.a.s.l., or in the vegetation immediately behind the North and East Beaches. In each case, about 2 cm of tail was removed and stored in 95% ethanol. Henderson samples were obtained as follows:

(i) Pre-eradication samples (henceforth pre-samples)—50 animals, September 2009.
(ii) Post-eradication samples (henceforth post-samples)
  (a) 1 animal, May 2012,
  (b) 63 animals, November 2012, and
  (c) 19 animals, August 2013.

The density of the vegetation across the island, coupled with the considerable cost of keeping a large vessel, two helicopters and personnel on station, meant that island-wide monitoring for rats surviving after the August 2011 bait drop was simply impractical. These twin problems, remoteness and onshore conditions, are likely to preclude sustained post-drop monitoring at many islands of high conservation value where eradication may be under consideration for the future.

To test the possibility that the continuing presence of rats on Henderson is not due to an introduction after the eradication attempt, samples were obtained from other islands. The nearest island to Henderson, and therefore the most likely source of a rat re-introduction, is Pitcairn, approximately 200 km to the southwest. Pitcairn rat samples (*n* = 30, July 2011; *n* = 18, June 2014) were obtained and preserved as for the Henderson samples in July 2011. Additional samples were obtained from more distant island groups, namely the Gambier archipelago in southeast French Polynesia 600 km west of Henderson (*n* = 38 successful genotypings from 26 rats obtained in April 2010 and 23 in January 2013), and the Cook Islands 3000 km west of Henderson (*n* = 10 amplified from Anchorage and Motu Tou islets off Suwarrow, May 2013).

### 2.2. Laboratory analysis

DNA was extracted from most tail-tips using a glass milk extraction method, adapted for use in microtitre plates rather than individual tubes. Briefly, tissue was digested in proteinase K and the liberated DNA adsorbed to flint glass particles in the presence of a 3× excess of 6 M NaI. After two ethanol washes, the DNA was eluted in 100 µl of low TE buffer. In a brief study commissioned from Landcare Research, New Zealand, to assess whether the single rat caught post-eradication in May 2012 was a re-introduction, samples from this individual, from 30 pre-eradication Henderson rats, and from 30 Pitcairn rats were extracted and analysed for eight microsatellite markers. These DNA samples were used by us without re-extraction.

Samples were genotyped at 19 microsatellite loci developed from the *Rattus norvegicus* genome (table 1). The 19 included seven of the eight loci used by Landcare, extended by testing a further 31 markers from *R. norvegicus* on *R. exulans*. In order to minimize the probability of linkage between microsatellites, the markers chosen for testing were selected from throughout the *R. norvegicus* genome. Genotyping was conducted in 10 µl multiplex PCR reactions (Qiagen® Multiplex PCR Kit, UK) with fluorescent-labelled forward primers containing approximately 20 ng DNA, following the manufacturer's protocol. Three sets of multiplex reactions were carried out, each with between five and seven primer pairs (table 1). PCR conditions comprised an initial denaturing step of 95°C for 15 min, followed by 35 cycles of 94°C denaturization for 30 s, 57°C annealing for 90 s and 72°C extension for 60 s, followed by a final extension of 30 min at 60°C. Following PCR, 1 µl of PCR product was mixed with 10 µl of loading mix (1 ml Hi-Di™ Formamide plus 20 µl of Genescan LIZ 500 ladder (ABI)) before visualization on an ABI3730 DNA Analyzer.

**Table 1.** A summary of the 19 microsatellite loci, amplified in three multiplex PCR reactions, used to genotype Pacific rat *Rattus exulans* samples. All primer sequences are available in the Rat Genome Database [14].

locus	fluorescent label	multiplex number	product size range (bp)	no. alleles
D7Arb16	HEX	1	87–104	9
D2Rat234	HEX	1	114–121	4
D8Mgh4	6-FAM	1	114–156	9
D12Rat36	HEX	1	182–195	8
D19Rat75	6-FAM	1	195–207	6
D10Rat20	6-FAM	2	97–128	12
D9Mit3	HEX	2	98–109	6
D11Rat7	HEX	2	131–149	5
D6Rat99	6-FAM	2	133–158	10
D5Rat83	6-FAM	2	166–180	6
D7Rat13	HEX	2	201–209	3
D15Rat77	6-FAM	2	240–261	11
D2Rat312	HEX	3	94–112	9
D17Mgh1	6-FAM	3	112–136	13
D1Rat313	6-FAM	3	153–167	8
D4Rat106	HEX	3	155–178	7
D6Rat100	6-FAM	3	174–186	7
D8Rat162	HEX	3	202–215	6
D14Rat39	6-FAM	3	223–257	14

### 2.3. Statistical analysis

Alleles were called using GeneMapper v. 3.7. The Henderson pre-bottleneck data were tested for the presence of null alleles using Cervus [15], with two having low frequency nulls (3% for D15Rat77 and 7% for D7Rat13). Another (D2Rat234) had an inferred null allele frequency of 36% and was excluded from all the analyses we report, which are therefore based on 18 loci. To explore the level of differentiation seen among our sampling regions and potentially exclude other neighbouring islands as a source of a re-introduction, we used the program Structure [16], with burn-in length 20 000 and 50 000 steps, varying the possible number of putative sub-groups (k) from one to 15 with 10 repetitions of each. We did not use sampling locality priors, allowed admixture and assumed that loci were unlinked. In addition, for the two focal islands, Henderson and Pitcairn, we constructed a joint individual-based neighbour-joining tree using a pairwise relatedness matrix, constructed following the methods of Queller & Goodnight [17]. The tree was calculated using Phylip [18].

Classical methods of estimating the effective population size (*N*_e_) of a bottleneck may struggle in our study. First, bottleneck size can be estimated through the associated loss of heterozygosity. However, this offers a rather blunt tool because detectable losses of heterozygosity require either very low numbers of survivors (less than 10) or a longer duration (i.e. no immediate recovery). Further, loss of heterozygosity could conceivably be distorted by the preferential survival of more heterozygous individuals [19]. Some other methods exploit features of the way microsatellites evolve and compare observed test statistics with those based on simulations assuming a strict stepwise mutation model (SMM) or related models. For example, the program ‘Bottleneck’ [20] is based on transient changes in the ratio of allele range to heterozygosity associated with the loss of rare alleles. However, the SMM is over-simple because it is now known that microsatellite mutations are centrally directed, with long alleles contracting and short alleles expanding. Moreover, cross-species markers can show strong departures from the SMM due to interruption mutations within the microsatellite itself [21]. Finally, our cross-species markers include several that may or actually do carry non-amplifying alleles.

To address these issues and to estimate the likely size of the bottleneck, we used two approaches. First, we used the program NeEstimator v. 2 [22] to implement the standard temporal method [23]. Second, we used stochastic simulations implemented using custom code written in Visual Basic (electronic supplementary material). For the latter, semi-realistic allele frequency distributions are established by allowing microsatellites to evolve under a strict SMM in a homogeneous population of size 1000 diploid individuals with mutation rate chosen such that heterozygosity was similar to that seen in our empirical data for pre-eradication attempt rats on Henderson. Each population is then reduced to bottleneck size *X* (*X* = 5–100, with equal sex ratio) and then allowed to expand rapidly at threefold per generation over a maximum of six generations back to a maximum size of 1000. During this realistically short time period, the contribution of new mutations can safely be ignored. Samples of 50 and 83 were drawn at random both immediately before the bottleneck and at the end of the simulation and used to assess heterozygosity, calculated as expected heterozygosity at Hardy–Weinberg equilibrium, and allele number. All simulations were repeated 100 times to obtain a mean and standard deviation.

As mentioned above, changes in heterozygosity are probably too slight to be informative. Consequently, we focused on the average change in frequency of individual alleles [24]. There are two potential issues with this approach. First, our estimated allele frequencies are based on modest samples of an unknown underlying distribution. Second, even if we did know the underlying frequency distributions, the chance that these are replicated closely during simulation is remote. Consequently, we systematically explored the fate of single alleles at all possible frequencies. Specifically, individual populations of 1000 individuals were founded carrying just two alleles (frequency of minor allele = 0.01–0.5, step 0.01). The bottleneck, recovery and sampling were performed as above, yielding a complete spectrum of frequency trios: unobserved true pre-bottleneck, pre-bottleneck in a sample the size we collected and post-bottleneck of the size we collected with, on average, 100 replicates of each observed pre-bottleneck frequency. Using these data, we can then translate our empirically observed allele frequencies into the expected average change in frequency for a bottleneck of any given size. To explore the impact of null alleles, all simulations were repeated two further times, adding null alleles to the pre-bottleneck population at the observed frequencies seen at two loci of (3% and 7%).

### 2.4. Testing post-eradication pesticide resistance

To address the question of whether the rats surviving on Henderson were resistant to brodifacoum, 58 rats were captured in July and August 2013 using Elliot live traps. On capture the weight and sex of each rat were recorded prior to placing them in individual 70 × 50 × 50 cm cages, made from wire mesh and plywood board. Each cage contained a food bowl and water dispenser, while plastic screens placed between cages prevented visual interaction.

Each rat had 3 days of acclimatization on a diet of guinea pig food (a non-toxic food source obtained from a pet food supplier; Animates Rabbit and Guinea Pig Food, Animates, Wellington, New Zealand). After the 3 days, each rat was re-weighed (to ensure it was healthy and not losing body mass) and randomly assigned to one of eight trial groups. There were a control group (no poison) and seven experimental groups exposed to progressively higher concentrations of brodifacoum in their diet. Each group had eight rats except for the two groups receiving the highest dose, where *n* = 5.

Each trial group was then presented with brodifacoum-laced peanut butter (brodifacoum 0.002% wet weight), weighed to deliver the dose of brodifacoum required by the rat's weight, along with some guinea pig food (table 2). The control group animals each received 1 g of non-toxic peanut butter. All rats had consumed all the poison within 24 h. After toxicant consumption, the rats’ diet returned to guinea pig food. Each rat was monitored twice daily until death or 14 days after the poison dose was ingested. Any rat clearly suffering and close to death was euthanized by cervical dislocation (this occurred twice); rats surviving after 14 days were released close to where captured.

**Table 2.** The eight trial groups and the dose of brodifacoum presented in mg/kg of rat body weight (bw). Each dose was calculated individually for each rat based on its post-acclimatization weight.

poison dose (mg/kg bw)	no. rats	number surviving
0	8	8
0.025	8	7
0.05	8	5
0.1	8	1
0.2	8	0
0.35	8	0
0.55	5	0
0.80	5	0

## 3. Results

### 3.1. Genetic substructure

**Figure 1.**
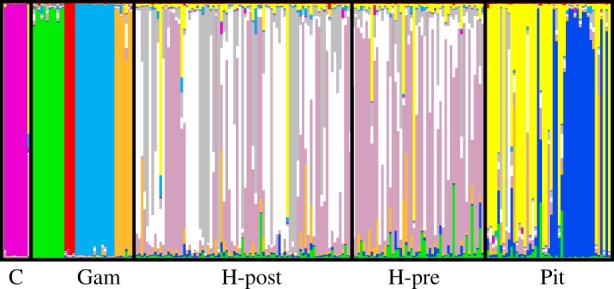
Structure plot of rats sampled from the Cook Islands (C), the Gambier archipelago (Gam), Henderson Island pre‐ and post‐eradication attempt, and Pitcairn (Pit).

The program Structure revealed the best-fit number of sub-groups to be 10 (electronic supplementary material, figure S2). Group membership is summarized in a bar plot (figure 1). This reveals a number of interesting features. First, rats from the Cook Islands and from four sampling localities within the Gambier archipelago are all assigned to their own groups with near 100% confidence and minimal overlap with either Henderson or Pitcairn (N.B. one Cook Islands sample came from a different region from the others and was clearly genetically different, failing to place in any of the 10 groups). Despite being genetically very similar ‘by eye’, in the sense that the same alleles occur in both samples at ostensibly similar frequencies, Pitcairn and Henderson samples are also largely separable. Within the islands, the two main groups on Pitcairn (yellow and blue) correspond well with the two different sampling efforts, while there are three main groups on Henderson (pink, white and grey). On Henderson, there is a weaker but still rather clear separation between pre-eradication samples (mainly pink) and post-eradication samples (largely white and grey).

**Figure 2.**
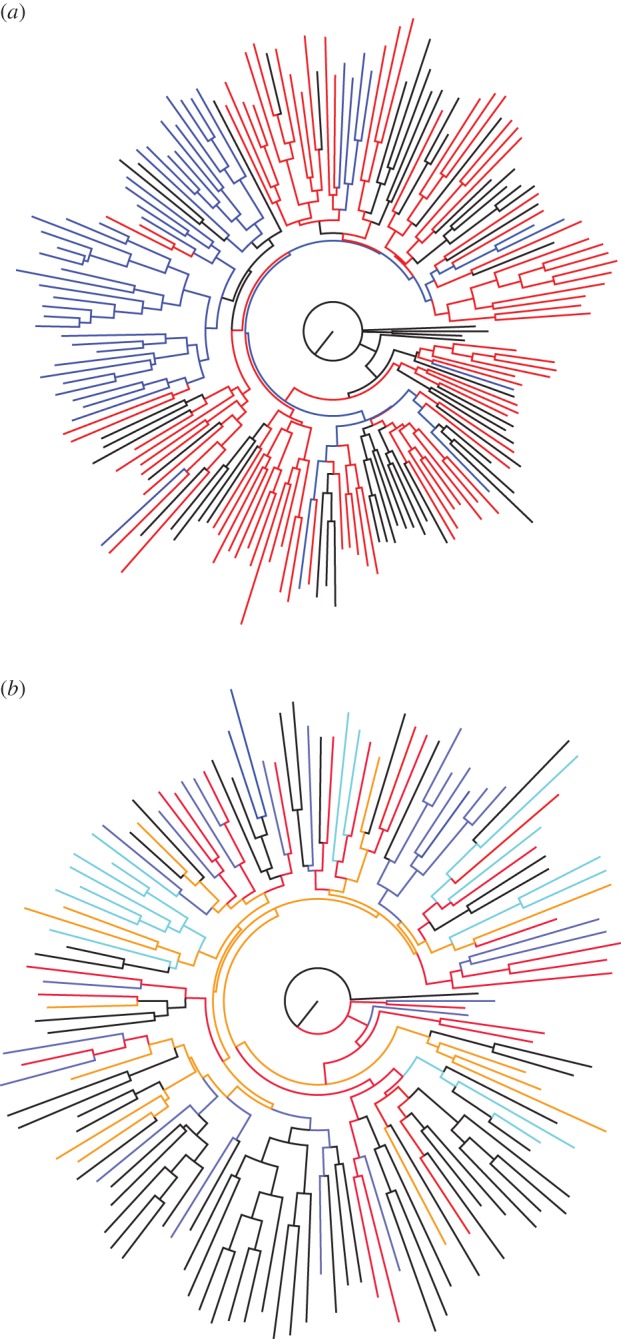
(*a*) Neighbour-joining tree of all Henderson and Pitcairn rats. The tree was constructed using a distance matrix of 1 – *R*, where *R* is relatedness *sensu* Queller and Goodnight. For clarity, individual rats are colour coded: blue, Pitcairn; black, Henderson pre-eradication attempt; red, Henderson post-eradication attempt. (*b*) The neighbour-joining tree of Henderson rats, according to trapping location and period. Red, North Beach pre-eradication; orange, North Beach post-eradication; dark blue, East Beach pre-eradication; light blue, East Beach post-eradication; black, plateau post-eradication.

The neighbour-joining tree of all Henderson and Pitcairn rats is shown in figure 2. Two important features are apparent. First, mirroring the Structure results, the Pitcairn rats fall mainly in a single, almost pure clade, indicating genetic separation (figure 2*a*). Second, the Henderson rats exhibit some degree of clustering, in that multiple rats from the same sampling location often cluster together (figure 2*b*). This may well be due to the sampling of related individuals on a scale of tens of metres. On the other hand, each of the primary sampling areas (E. Beach, N. Beach, Plateau; electronic supplementary material, figure S1) contributes clusters spread right across the tree, suggesting appreciable gene flow between the different regions. In addition, there is no strong tendency for pre- and post-eradication attempt samples to cluster strongly. We conclude that, while mild sub-structure is present, this is probably not strong enough to indicate strongly restricted gene flow between the island regions.

### 3.2. Estimating the likely minimum number of rats

**Figure 3.**
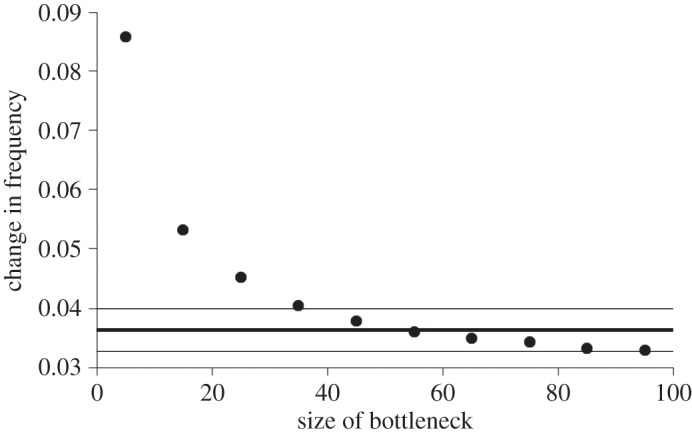
Change in allele frequency among Henderson rats. The heavy horizontal line shows the estimated average change in individual microsatellite allele frequency between rats sampled before and after the eradication programme. The errors on the estimate are one standard error of the mean, obtained by averaging across 18 loci. Solid dots represent the mean change in allele frequency of microsatellites when a simulated population passes through a bottleneck of the size given on the *x*-axis.

Unbiased heterozygosity reveals negligible change while expected heterozygosity actually increases slightly. However, as discussed in Material and methods, changes in overall heterozygosity provide a relatively crude measure and would require a very severe bottleneck to show a detectable change. Changes in allele frequency may provide a more robust estimate because each locus carries multiple alleles, each of which provide a point estimate. Using this approach, we find excellent agreement between three related estimators, implemented in NeEstimator v2, and our own stochastic simulations. In all cases, the best-fit number of survivors was an effective population size of approximately 50 ± 30 rats (table 3 and figure 3).

**Table 3.** Estimated bottleneck size based on the temporal method. The program NeEstimator v2 was used to estimate the likely size of the bottleneck caused by the attempted eradication of rats on Henderson Island. The program implements three related methods (Pollak [24], Nei & Tajima [25], Jorde & Ryman [26]) and sets the minimum allele frequency accepted to four thresholds (5%, 2%, 1% and 0%), with resulting number of alleles considered in parentheses. Resulting estimates of *N*_e_ are in bold with 95% confidence intervals in parentheses. Confidence intervals are obtained both as parametric approximations and by jackknifing across loci. We present the parametric estimates which are generally a little tighter, particularly for the Jorde/Ryman method.

minimum frequency	0.05 (61)	0.02 (75)	0.01 (85)	0 (97)
Pollak	**42.2** (23.5, 79.1)	**44.9** (26.1, 80.2)	**48.8** (29.0, 86.4)	**59.2** (35, 107.7)
Nei/Tajima	**45.6** (25.0, 87.7)	**47.4** (27.4, 86.3)	**50.8** (29.9, 91.0)	**59.7** (35.3, 109.0)
Jorde/Ryman	**35.8** (24.2, 49.6)	**35.3** (24.9, 47.4)	**35.1** (25.4, 46.5)	**35.3** (26.1, 45.0)

### 3.3. Toxicity tests

Mortality was 100% for dose levels of 0.2 mg kg^−1^ (bw) and above; only the control group had zero mortality (table 2). The median lethal dose LD_50_, calculated in R v. 3.0.1, was 0.061 ± 0.02 mg kg^−1^ (95% CI).

## 4. Discussion

We genotyped samples collected from other Pacific islands for 18 polymorphic microsatellites and found no evidence that re-introduction from these sources is the reason for the continuing presence of rats on Henderson Island. We therefore conclude that rats remain on Henderson because of a failure to eradicate. We then compared the microsatellite genotypes obtained from rats sampled before and after the eradication attempt, to estimate the number to which the population was reduced. Heterozygosity was unchanged but the average change in frequency of individual alleles suggests an effective population size (*N*_e_) of around 50 rats at the deepest part of the bottleneck.

Early studies of the relationship between effective population size (*N*_e_) and census size (*N*) suggested that most species have a ratio *N*_e_/*N* in the approximate range 0.25–0.75 [27]. A more recent study [28] suggests that mammals have an average ratio of 0.749 ± 0.117 s.d., implying that our estimate of *N*_e_ = 50 rats will translate into a census size of 58–80 rats.

An estimate of approximately 60–80 actual survivors seems plausible. Following the attempted eradication, no rats were seen over the first three months when fieldworkers were continuously present. Even with the dense vegetation, this seems unlikely unless the numbers left alive were very small. On the other hand, once the first rat was seen by a temporary visitor seven months after the bait-drop, numbers did rebuild rapidly [10], something that seems less likely if the population had been reduced below 10. Under such circumstances inbreeding depression and the difficulty of finding a mate might have slowed recovery [29].

Our analysis reveals subtle population substructure but probably not enough to impact our conclusions. Thus, Pitcairn was sampled at two different locations and times and these are reasonably well resolved by Structure (figure 1). Similarly, while the pre-eradication sample from Henderson appears fairly homogeneous, Structure suggests three weak groups among animals sampled after the eradication attempt. These groups may well reflect a small number of pockets of survivors which interbred before dispersing more widely. On the other hand, the individual-based tree shows no obvious relationship with geography beyond the presence of small, possibly family clusters (figure 2), and Structure places some Pitcairn rats on Henderson and *vice versa*. Importantly, appreciable substructure should result in weak homozygote excess across loci and we find a weak heterozygote excess (data not shown).

Logistic constraints meant we did not sample rats across the full extent of Henderson (electronic supplementary material, figure S1). However, we did sample from sites that are reasonably distant in the context of the entire island and find very little (arguably negligible) structure beyond that which might be expected from the sampling of relatives. Moreover, Pitcairn shows only marginal distinctiveness. If the Henderson recovery was based on immigration from Pitcairn, presumably by less than 10 individuals, we would expect extremely low diversity, significantly lower than we observe, and the post-eradication rats should form a reasonably distinct sub-clade within the Pitcairn clade. That post-eradication rats have higher diversity and co-cluster with pre-eradication Henderson rats therefore argues strongly that this is survival rather than re-introduction. Combined, this suggests that our sampling has captured the majority of Henderson diversity: it would be truly remarkable if an unsampled clade existed on Henderson that was more different than rats from Pitcairn.

Our toxicity tests revealed that only the control rats receiving a zero brodifacoum dose showed no mortality. The calculated LD_50_ of 0.061 mg kg^−1^ among rats on Henderson in 2013 was considerably lower than the acute LD_50_ of 0.32 mg kg^−1^ found by Conor & Booth [30] in another population of *R. exulans*. These results therefore provide no support for the possibility that failure of the 2011 operation was due to brodifacoum resistance among the Henderson rats.

Since 0.2 mg kg^−1^ (or higher) was lethal for all rats tested, an average rat of 80 g (own data) would certainly die after ingesting 0.016 mg of toxin. Given that the active toxin, brodifacoum, constituted 0.002% (20 mg kg^−1^) of pelleted bait weight in the 2011 eradication attempt [8], the fatal amount of toxicant would be ingested by a rat eating 0.8 g of bait. Since each bait pellet weighed roughly 1.6 g [11], an average weight rat would die after consuming less than a single pellet. The amount of bait broadcast on the island, averaging almost 20 kg ha^−1^, was equivalent to hundreds of pellets per rat.

Our genetic results show that the eradication attempt was very nearly successful, and the brodifacoum tests provide no evidence of resistance to the toxicant used. The genetic results also argue against any re-introduction which is in any case highly unlikely given our knowledge of inter-island boat movements. It appears then that failure was caused neither by a tiny number of survivors nor by several hundred survivors, the latter indicating some systematic fault in the baiting approach used. Rather, the failure could have been due to a small number of rats either not encountering poison pellets or showing a preference for natural food over bait pellets. In tropical or sub-tropical latitudes, it may always be more difficult than in temperate latitudes to ensure that operations are undertaken when the availability of natural food is very low [31], especially when critical ‘postpone or proceed’ decisions have of necessity to be taken months before the operation. Further plant phenological studies that enhance understanding of the ebb and flow of natural food on Henderson would be useful [32]. Arguably even more useful and of widespread value to other projects would be work directed towards increasing the attractiveness of bait pellets over natural food [33].

## Supplementary Material

First file Figure S1 Outline map of Henderson Island showing trapping areas behind the island's beaches and on the raised plateau. Figure S2. Determining group number, K. The replicate STRUCTURE runs were conducted for each value of K from one to 15. The graph shows + one standard error of log likelihood.

## Supplementary Material

Second file Genetic data

## Supplementary Material

Third file Annotated Visual Basic code for simulating impact of bottlenecks

## Data accessibility

See the electronic supplementary material.
