# Supplementary material for: Rat eradication comes within a whisker! A case study of a failed project from the South Pacific
Source: R Soc Open Sci. 2016 Apr 20;3(4):160110. doi: 10.1098/rsos.160110 (PMC4852649; doi:10.1098/rsos.160110)
Supplement: First file Figure S1 Outline map of Henderson Island showing trapping areas behind the island's beaches and on the raised plateau. Figure S2. Determining group number, K. The replicate STRUCTURE runs were conducted for each value of K from one to 15. The graph shows + one standard error of log likel [file rsos160110supp1.doc]

Supplementary Figures Amos et al.

**Figure S1** Outline map of Henderson Island showing trapping areas behind the island’s beaches and on the raised plateau.

0 km 2 2

North Beach trapping area

East Beach

trapping area

Grid of plateau paths

used for trapping

**N**

Path linking E

& N Beaches

**Figure S2.** Determining group number, K. The replicate STRUCTURE runs were conducted for each value of K from one to 15. The graph shows + one standard error of log likelihood.
