# Supplementary material for: Rat eradication comes within a whisker! A case study of a failed project from the South Pacific
Source: R Soc Open Sci. 2016 Apr 20;3(4):160110. doi: 10.1098/rsos.160110 (PMC4852649; doi:10.1098/rsos.160110)
Supplement: Third file Annotated Visual Basic code for simulating impact of bottlenecks [file rsos160110supp3.doc]

Annotated Visual Basic code for simulating impact of bottlenecks of different sizes on allele frequencies assuming samples of size 50 and 82 before and after the even.

Dim Mu As Single, Het As Single, Nal As Integer, silent As Integer, none As Integer
Dim popsize As Long, Leadin As Integer, w1 As Integer, w2 As Integer
Dim A(1000, 2, 2) As Integer, counts(2, 2) As Integer, ptge As Integer
Dim Survive As Integer, Initial As Integer, n1, n2 As Long

Sub Main()
    Worksheets("nulls2").Activate
    rw = 0
    Randomize Timer
    popsize = 1000 ' set the default population size of 1000
    For Survive = 5 To 100 Step 10 ' explore a range of survivor numbers
        For ptge = 1 To 50
            For rep = 1 To 1000
                rw = rw + 1
                For silent = 0 To 3
                    Setup ' initialise the genotypes
                    Phase1 ' two generations of reproduction at full population size
                    Call Assess(1) ' calculate pre-bottleneck frequncies
                    prefq1 = counts(1, 1) / (2 * (50 - none1))
                    prefq2 = counts(2, 1) / (2 * (50 - none1))
                    Het1 = Het: Nal1 = Nal: none1 = none ' store parameters
                    bottle ' undergo a bottleneck of size survive
                    Call Assess(2) ' calculate post-bottleneck frequencies
                    Het2 = Het: Nal2 = Nal: none2 = none ' store prost-bottleneck paramters
                    postfq1 = counts(1, 2) / (2 * (82 - none2))
                    postfq2 = counts(2, 2) / (2 * (82 - none2))
                    
                    If prefq1 < prefq2 Then ' store results for minor allele
                        pre = prefq1
                        Post = postfq1
                    Else
                        pre = prefq2
                        Post = postfq2
                    End If
                    
                    Cells(rw, 1 + 4 * silent) = Survive ' output results
                    Cells(rw, 2 + 4 * silent) = pre
                    Cells(rw, 3 + 4 * silent) = Post
                    Cells(rw, 4 + 4 * silent) = Abs(pre - Post)
                Next silent
            Next rep
        Next ptge
    Next Survive
    Average ' calculate average values for all pre-bottleneck frequencies across all bottleneck sizes
End Sub

Sub Assess(x As Integer) ' count allele frequencies etc.
    If x = 1 Then num = 50 Else num = 82 ' used emprical sample sizes
    Nal = 0 ' zero allele count
    Het = 1 ' set het to 1
    Erase counts ' zero allele counter
    none = 0 ' zero null genotype counter
    n = 0
    
    For g = 1 To num 'sample pre-number of rats
        ' variable 'nulls' = 1 if second allele is null and 2 if first allele is a null
        nulls = Abs(2 * (A(g, 1, w2) = 3)) + Abs(1 * (A(g, 2, w2) = 3))
        For h = 1 To 2 ' assay each allele
            If nulls = 0 Then ' standard genotype
                counts(A(g, h, w2), x) = counts(A(g, h, w2), x) + 1
            ElseIf nulls < 3 Then ' heterozygote null
                counts(A(g, nulls, w2), x) = counts(A(g, nulls, w2), x) + 1
            Else ' homozygote nulls
                none = none + 0.5
            End If
        Next h
    Next g
    
    For h = 1 To 2 ' scan both alleles visibles
        If counts(g, x) > 0 Then
            Nal = Nal + 1 ' store allele number
            Het = Het - (counts(h, x) / ((num - none) * 2)) ^ 2 ' and heterozygosity
        End If
    Next h
End Sub

Sub bottle()
    For f = 1 To popsize ' zero the recipient array, just to be sure
        A(f, 1, w2) = 0
        A(f, 2, w2) = 0
    Next f
    
    For gen = 0 To 20
        n1 = Survive * 3 ^ gen ' exponential increase up to a maximum of popsize (1000)
        n2 = Survive * 3 ^ (gen + 1)
        If n1 > popsize Then n1 = popsize
        If n2 > popsize Then n2 = popsize
        fem = Int(n1 / 2) ' introduce sexes because important at small sizes
        mal = n1 - fem
        For f = 1 To n2
            Z = Int(Rnd(1) * fem) + 1 ' select first half female
            If Rnd(1) < 0.5 Then A(f, 1, w2) = A(Z, 1, w1) Else A(f, 1, w2) = A(Z, 2, w1)
            Z = Int(Rnd(1) * mal) + fem ' select second half male
            If Rnd(1) < 0.5 Then A(f, 2, w2) = A(Z, 1, w1) Else A(f, 2, w2) = A(Z, 2, w1)
        Next f
        If n1 = popsize Then Exit For ' terminate when popsize is reached (drift will be minimal afterwards)
        w1 = 3 - w1
        w2 = 3 - w2
    Next gen
End Sub

Sub Phase1() ' two generations to randomize the genotypes (only 1 really necessary!)
    For f = 1 To 2
        Reproduce
        w1 = 3 - w1 ' change the array pointers
        w2 = 3 - w2
    Next f
End Sub

Sub Reproduce()
    For g = 1 To popsize
        Z = Int(Rnd(1) * popsize) + 1
        If Rnd(1) < 0.5 Then A(g, 1, w2) = A(Z, 1, w1) Else A(g, 1, w2) = A(Z, 2, w1)
        Z = Int(Rnd(1) * popsize) + 1
        If Rnd(1) < 0.5 Then A(g, 2, w2) = A(Z, 1, w1) Else A(g, 2, w2) = A(Z, 2, w1)
    Next g
End Sub

Sub Setup()
    If silent < 3 Then times = 3 Else times = 9
    Erase A
    m = Int(Rnd(1) * 20) + 1
    ' w1 and w2 are pointers that alternate each generation between 1 and 2.  Thus, genotypes in
    ' generation x stored in w1 are used to populate generation x+1 in w2.  The pointers are then
    ' reversed.
    w1 = 1
    w2 = 2
    x = 0
    For f = 1 To popsize ' initialise all individuals with genotype 1,1
        A(f, 1, 1) = 1
        A(f, 2, 1) = 1
    Next f
    
    ppn = ptge * popsize / 100 ' set the initial target percentage frequency of the minor allele
    For g = 1 To ppn ' set that frequency of individuals to genotype 2,2
        A(g, 1, 1) = 2
        A(g, 2, 1) = 2
    Next g
    ppn2 = times * silent * popsize / 100 ' if null alleles are present, add these
    For g = ppn + 1 To ppn + ppn2
        A(g, 1, 1) = 3
        A(g, 2, 1) = 3
    Next g
    
End Sub

Sub Average()
    Dim sm(20, 100, 2) As Single ' array for storing averages
    For silent = 0 To 3
        Erase sm
        f = 1
        Do
            v1 = (Cells(f, 1 + silent * 4) + 5) / 10 ' bottleneck size
            v2 = Cells(f, 2 + silent * 4) * 100 ' minor allele frequency pre-bottleneck
            v3 = Cells(f, 4 + silent * 4) ' change in frequency
            sm(v1, v2, 1) = sm(v1, v2, 1) + v3
            sm(v1, v2, 2) = sm(v1, v2, 2) + 1
            f = f + 1
        Loop Until IsEmpty(Cells(f, 1 + silent * 4))
        ' then output the averages
        For g = 1 To 10
            For h = 0 To 100
                If sm(g, h, 2) > 1 Then Cells(h + 2, g + 20 + silent * 11) = sm(g, h, 1) / sm(g, h, 2)
            Next h
        Next g
    Next silent
End Sub
